# Performance of Machine Learning for Tissue Outcome Prediction in Acute Ischemic Stroke: A Systematic Review and Meta-Analysis

**DOI:** 10.3389/fneur.2022.910259

**Published:** 2022-07-08

**Authors:** Xinrui Wang, Yiming Fan, Nan Zhang, Jing Li, Yang Duan, Benqiang Yang

**Affiliations:** ^1^Department of Radiology, General Hospital of Northern Theater Command, Shenyang, China; ^2^Department of Orthopedics, Chinese PLA General Hospital, Beijing, China; ^3^Department of Radiology, Changhai Hospital, Shanghai, China

**Keywords:** ischemic stroke, machine learning, deep learning, computed tomography, magnetic resonance imaging, meta-analysis

## Abstract

Machine learning (ML) has been proposed for lesion segmentation in acute ischemic stroke (AIS). This study aimed to provide a systematic review and meta-analysis of the overall performance of current ML algorithms for final infarct prediction from baseline imaging. We made a comprehensive literature search on eligible studies developing ML models for core infarcted tissue estimation on admission CT or MRI in AIS patients. Eleven studies meeting the inclusion criteria were included in the quantitative analysis. Study characteristics, model methodology, and predictive performance of the included studies were extracted. A meta-analysis was conducted on the dice similarity coefficient (DSC) score by using a random-effects model to assess the overall predictive performance. Study heterogeneity was assessed by Cochrane *Q* and Higgins *I*^2^ tests. The pooled DSC score of the included ML models was 0.50 (95% CI 0.39–0.61), with high heterogeneity observed across studies (*I*^2^ 96.5%, *p* < 0.001). Sensitivity analyses using the one-study removed method showed the adjusted overall DSC score ranged from 0.47 to 0.52. Subgroup analyses indicated that the DL-based models outperformed the conventional ML classifiers with the best performance observed in DL algorithms combined with CT data. Despite the presence of heterogeneity, current ML-based approaches for final infarct prediction showed moderate but promising performance. Before well integrated into clinical stroke workflow, future investigations are suggested to train ML models on large-scale, multi-vendor data, validate on external cohorts and adopt formalized reporting standards for improving model accuracy and robustness.

## Introduction

Stroke is a life-threatening disease accounting for approximately 10% of all deaths and presenting an estimated lifetime risk of 25% worldwide (1). Recanalization of the occluded vessels is the only effective treatment to restore blood flow and prevent neural functional deterioration. Early studies suggested 4.5 and 6 h as the time window for intravenous thrombolysis (IVT) and endovascular thrombectomy (EVT) from symptoms onset (2–4). Recent advances in endovascular approaches have broadened the boundaries of eligible patient selection and expanded the time window to 24 h by using advanced neuroimaging (5, 6).

Currently, acute stroke imaging allows estimating the ischemic core and penumbra by predefined imaging thresholds. An apparent diffusion coefficient threshold between 600 and 625 × 10^−3^ mm^2^/s remains a robust parameter for infarct core estimation, and a decreased relative cerebral blood flow (rCBF) threshold of <30% has been extensively used to quantify final infarct size for CT-based method. The mismatch between infarct core and perfusion deficit identified by time to maximum of the residue function (Tmax) with a delay >6s provides a delineation of tissue at risk (7, 8). Despite the easy application of using single-valued thresholds to predict ischemic tissue outcome, conventional thresholds derived from approximate linear statistic models would probably fail to capture the heterogeneity of stroke lesion development from baseline imaging. Moreover, thresholds based on a single imaging modality disregarded the complementary effect of multimodal imaging, thus limiting the reliability in delineating infarct lesions.

Recent advances in machine learning (ML) offer promising applications in medical imaging by learning informative features and patterns from structured input data. It also drives the emergence of deep learning (DL) subfield, which has shown impressive results in medical image processing without prior selection for relevant features (9, 10). Given the suboptimal performance of the conventional thresholding methods, initial studies attempted to apply ML and DL-based approaches and showed clear advantages for more precise prediction of the final infarct lesion from baseline imaging (11–17). These promising results inspired investigators to propose novel model methodologies by improving algorithm architectures, combining multi-modality input parameters, and applying in different clinical scenarios.

Although studies on this topic are growing, there is a lack of studies that review the general applications of the state-of-the-art ML-based approaches in ischemic core estimation. Therefore, we conducted this systematic review and meta-analysis to provide an overview of the potential advantages and remaining challenges of ML-based model methodologies for final infarct lesion prediction from acute stroke imaging, evaluate the overall performance of existing approaches, and provide suggestions for future research to potentially aid in acute ischemic stroke (AIS) management.

## Methods

This systematic review and meta-analysis was performed following the Preferred Reporting Items for Systematic Reviews and Meta-Analyses (PRISMA) statement (18).

### Literature Search and Study Selection

We comprehensively searched PubMed, EMBASE, Cochrane Library, Science Direct, Springer, and IEEE Xplore Digital Library databases from inception to May 31, 2022, with the following keywords: “machine learning”, “deep learning”, “neural network”, “stroke”, “cerebrovascular event”, “cerebral infarct”, “computed tomography”, “magnetic resonance imaging”. Studies that developed ML algorithms for predicting the final infarct lesion from baseline acute stroke imaging were included. Eligible studies satisfying the following inclusion criteria were included in the meta-analysis: (1) study cohort was AIS patients; (2) study described ML algorithms for predicting ischemic core tissue from baseline CT or MR imaging; (3) reference standard (i.e., ground truth) was true infarct lesion segmented on follow-up imaging; (4) prediction performance was reported as dice similarity coefficient (DSC) score; (5) imaging sets for algorithm training and test were clearly defined; (6) published articles with full text; and (7) English language articles. Review articles, conference abstracts, letters, case reports including fewer than 10 patients, and non-human research were excluded. If studies came from the same cohort or compared different algorithms on the same dataset, we only retained the article with the largest sample size or the best-performing algorithm in the quantitative synthesis in case sample duplicate or overlapping would affect the overall pooled effect size.

One investigator (XW) read the titles and abstracts of all records. After preliminary screening, potentially eligible articles were shortlisted. Two investigators (XW and YF) independently read the full-text articles to assess eligibility, with disagreements resolved by discussion and consensus.

### Data Extraction and Quality Assessment

Two investigators (XW and YF) independently extracted data from the included studies using a predefined data extraction sheet. Disagreements were re-evaluated and determined by a third investigator (NZ). The extracted data included: (1) first author and year; (2) source of the dataset; (3) sample size including the total patient number and numbers of the training, validation, and test sets; (4) model methodology, including algorithm types, input parameters and standard reference; (5) predictive performance, including the primary performance metric of DSC score and secondary metrics of area under the receiver operating characteristic curve (AUC), sensitivity, specificity, accuracy, precision, recall and volume error between the prediction result and the standard reference.

To assess the quality of ML-based diagnostic accuracy studies, Collins and Moons initially introduced a modified version of the Transparent Reporting of a Multivariable Prediction Model for Individual Prognosis or Diagnosis statement specific to machine learning (TRIPOD-ML) (19). However, the TRIPOD-ML guideline was complicated and covered a broad range of ML applications. The Radiology editorial board has developed a list of nine key considerations to improve the soundness and applicability of artificial intelligence research in diagnostic imaging (20). We adapted these items as quality assessment criteria in our study. Two investigators (XW and YF) independently evaluated the risks of bias using this questionnaire, with disagreements resolved by discussion and consensus.

### Statistical Assessment

We estimated the overall performance of the ML models by using the DSC score, a commonly used volume-based performance metric for target segmentation. The DSC score represents the overlap between the prediction segmentation and the standard reference, ranging from 0 (indicates no overlap) to 1 (indicates complete overlap). For effect size calculation in the meta-analysis, the mean DSC score with standard deviation (SD) or 95% confidence interval (CI) was required. When study reported the DSC score as median and interquartile range (IQR), the mean and SD was converted using a quantile estimating method described by Wan et al. (21). The sample mean ($\bar{X}$) and SD (*S*) were estimated as follows, where *q*1 referred to the first quartile, *m* referred to the median, and *q*3 referred to the third quartile.


$$\begin{array}{l} \bar{X}\;\approx \frac{q1+m+q3}{3}\text{ and S }\approx \frac{q3-q1}{1.35} \end{array}$$

A random-effects model meta-analysis was performed, and forest plots were generated to depict the effect size of individual studies and overall performance. The heterogeneity across the included studies was assessed using the Cochrane *Q* and Higgins *I*^2^ tests, where the *p-*value <0.05 in Cochrane *Q* test and Higgins *I*^2^-value > 75% indicated significant heterogeneity (22). Due to the high heterogeneity observed in this study, sensitivity analysis using the one-study removed method was conducted to explain the heterogeneity of the results. Subgroup analyses were performed according to algorithm types (conventional ML classifiers and deep neural networks) and imaging modality for model input (CT and MR data). Publication bias was examined by creating a funnel plot and Egger's bias test (23). Statistical analyses were performed using the STATA 17.0 statistical package (StataCorp, Stata Statistical Software). Two-sided *p*-value <0.05 was considered statistically significant.

## Results

**Figure 1:**
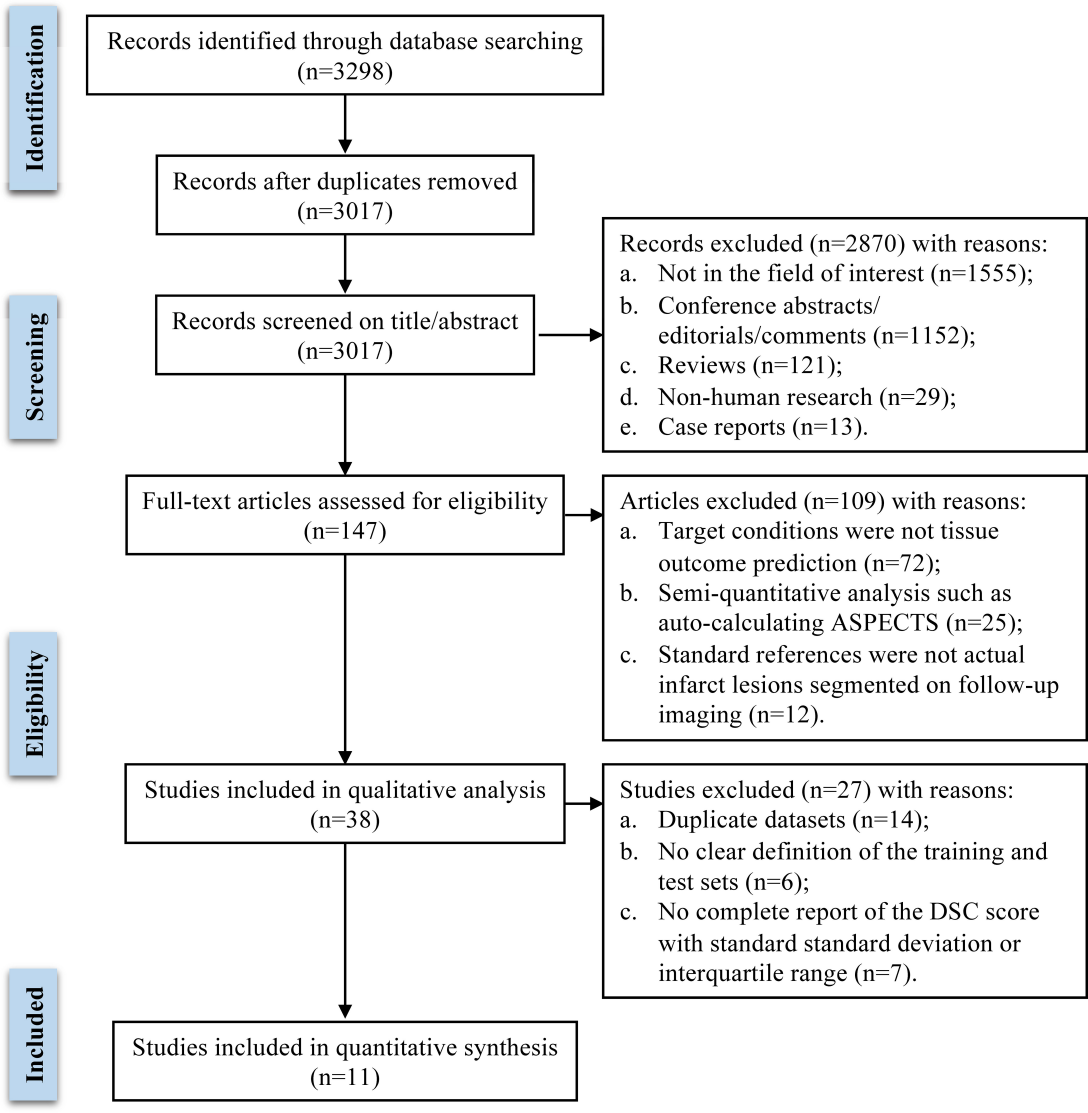
Flow diagram of literature review and study selection process.

A total of 3,298 publications were initially identified through database searching. After removing 281 duplicate records, the remaining 3,017 publications were screened preliminarily. Based on title and abstract, 2,870 articles were excluded, and 147 articles were assessed for eligibility by two investigators independently. After full-text review, 38 studies were included in the systematic review. Among them, 11 studies that met the inclusion criteria and provided sufficient quantitative data were included in the meta-analysis, and 27 studies were excluded for the following reasons: 14 studies proposed ML models trained and tested on duplicate datasets, 6 studies didn't clearly define the training and testing sets, 7 studies didn't report a complete DSC score with standard deviation or interquartile range. The literature search flow diagram is presented in Figure 1.

### Study Characteristics and Model Methodology: A Systematic Review

Based on full-text evaluation, 38 studies were included in the systematic review. Study characteristics, model methodology, and predictive performance are summarized in Table 1. Of the 38 included studies, 12 studies were based on single-center datasets (11, 12, 24, 25, 30, 34, 38, 41, 45–47, 52), and 26 studies were conducted on multicenter datasets, including 9 on registered clinical trials [13–17, 34–37] and 13 on two publicly available databases (Ischemic Stroke Lesion Segmentation [ISLES] challenges 2017 and 2018, http://www.isles-challenge.org) (27–29, 32, 37, 39, 40, 42, 44, 49–51, 53). All except two studies (45, 48) reported validation methods for the proposed model including using an independent test set, k-fold cross-validation, and leave-one-out cross-validation. External validation was only performed by one study (13).

**Table 1:** Study characteristics, model methodology, and predictive performance of the included studies.

**First author and year**	**Study characteristics**						
	**Dataset source**	**Inclusion/** **exclusion criteria**	**Total patient number (n)**	**Training and validation sets (n)**	**Test set (n)**	**External test set**	**Multivendor images**
Gottrup et al. (24)	Single center	N	14	Leave-one-out cross validation		N	NR
**McKinley et al**. **(****25****)**	Single center	Y	61	25	36	N	N
Livne et al. (26)	Multicenter (I-KNOW study and the Ischemic Preconditioning trial)	Y	195	≈156	≈39	N	Y
Nielsen et al. (14)	Multicenter (I-KNOW and remote ischemic preconditioning studies)	Y	222	187	35	N	Y
Pinto et al. (27)	Multicenter (ISLES 2017 dataset)	Y	75	43	32	N	N
Winzeck et al. (28)	Multicenter (ISLES 2017 dataset)	Y	75	43	32	N	N
Clèrigues et al. (29)	Multicenter (ISLES 2018 dataset)	Y	103	63	40	N	Y
Ho et al. (30)	Single center	Y	48	≈43	≈5	N	N
**Kasasbeh et al**. **(****31****)**	Multicenter	Y	103	≈82	≈21	N	NR
Pérez Malla et al. (32)	Multicenter (ISLES 2017 dataset)	Y	75	43	32	N	N
Robben et al. (33)	Multicenter (MR CLEAN study)	Y	188	≈150	≈38	N	NR
Winder et al. (34)	Single center	Y	90	Leave-one-out cross validation		N	N
Grosser et al. (35)	Multicenter	Y	99	Leave-one-out cross validation		N	NR
Grosser et al. (36)	Multicenter	Y	99	Leave-one-out cross validation		N	NR
Hu et al. (37)	Multicenter (ISLES 2017 dataset)	Y	75	43	32	N	N
**Kim et al**. **(****38****)**	Single center	Y	92 unsuccessful recanalization 36 and successful recanalization 56	53	39	N	N
Kumar et al. (39)	Multicenter (ISLES 2017 dataset)	Y	75	43	32	N	N
**Pinto et al**. **(****40****)**	Multicenter (ISLES 2017 dataset)	Y	75	43	32	N	N
Qiu et al. (41)	Single center	Y	257	157	100	N	N
Wang et al. (42)	Multicenter (ISLES 2018 dataset)	Y	103	63	40	N	Y
Yu et al. (17)	Multicenter (ICAS and DEFUSE-2 studies)	Y	182	≈146	≈36	N	NR
**Benzakoun et al**. **(****11****)**	Single center	Y	394	≈358	≈36	N	N
**Debs et al**. **(****43****)**	Multicenter (HIBISCUS-STROKE and I-KNOW cohorts)	Y	109 reperfused 74 and non-reperfused 35	Reperfused≈69 and non-reperfused≈28	Reperfused≈15 and non- reperfused≈ 7	N	NR
Hakim et al. (44)	Multicenter (ISLES 2018 dataset)	Y	103	63	40	N	Y
Hokkinen et al. (45)	Single center	Y	83	NR	NR	N	N
Hokkinen et al. (46)	Single center	Y	89	None	89	N	N
Klug et al. (47)	Single center	Y	144 intravenous thrombolysis (IVT) 80, endovascular thrombectomy (EVT) 64	≈115	≈29	N	N
**Kuang et al**. **(****13****)**	Multicenter (Prove-IT study and HERMES collaboration)	Y	205	68	137	Y	NR
Modrau et al. (48)	Multicenter (TEA-Stroke Trial)	Y	52 theophylline 27 and control group 25	NR	NR	N	NR
Pinto et al. (49)	Multicenter (ISLES 2017 dataset)	Y	75	43	32	N	N
Qiu et al. (15)	Multicenter (Prove-IT study)	Y	196	170	26	N	NR
**Soltanpour et al**. **(****50****)**	Multicenter (ISLES 2018 dataset)	Y	103	63	40	N	Y
Vupputuri et al. (51)	Multicenter (ISLES 2017 dataset)	Y	75	43	32	N	N
**Yu et al**. **(****16****)**	Multicenter (ICAS, DEFUSE and DEFUSE-2 studies)	Y	185	118	67	N	NR
He et al. (12)	Single center	Y	70	59	11	N	N
**Lin et al**. **(****52****)**	Single center	Y	261	≈209	≈52	N	NR
Shi et al. (53)	Multicenter (ISLES 2018 dataset)	Y	103	63	40	N	Y
**Zhu et al**. **(****54****)**	Multicenter	N	89	≈71	≈18	N	N
	**Model methodology**			**Predictive performance**	
**First author and year**	**Summary of the model**	**Input parameters**	**Ground Truth**	**Primary metric** **(DSC score)**	**Secondary metrics**
Gottrup et al. (24)	k-nearest neighbor classification	MR-CBF, CBV, MTT, DWI, ADC, T2WI	Infarct lesions manually segmented on follow-up T2WI 5 days or later	NR	AUC: 0.814 ± 0.001 Sensitivity: 0.73 Specificity: 0.73
**McKinley et al**. **(****25****)**	Random forest classifier, including segmentation and predictive classifiers	Features extracted from MR-T1 contrast, T2WI, ADC, CBF, CBV, TTP, Tmax	Final infarct lesions manually segmented on follow-up T2WI at 90 days by 2 radiologists	0.34 ± 0.22	AUC: 0.94 ± 0.08 Sensitivity: 0.52 Specificity: 0.99 Precision: 0.56
Livne et al. (26)	Extreme gradient boosting (XGBoost)	MR-DWI, T2-FLAIR, and TTP derived from the concentration curve; CBF, MTT and Tmax using oscillatory singular value decomposition deconvolution; CBF, CBV, MTT, Tmax, relative transit time heterogeneity and capillary transit time heterogeneity using a statistical approach	Final infarct lesions semi-automatically segmented on follow-up T2-FLAIR	NR	AUC: 0.92 Accuracy: 0.84
Nielsen et al. (14)	Modified SegNet	MR-mean capillary transit time, CBV, CBF, cerebral metabolism of oxygen, relative transit time heterogeneity, delay, TRACE DWI, ADC, and T2-FLAIR	Infarcts lesions manually segmented on follow-up T2-FLAIR at 30 days by 4 expert radiologists	NR	AUC: 0.88 ± 0.12
Pinto et al. (27)	Fully convolutional U-Net combined with a 2D-dimensional gated recurrent unit layer	MR-ADC, rCBF, rCBV, MTT, TTP, Tmax and clinical information-TICI score	Final infarct lesions manually segmented on follow-up T2WI at 90 days by a neuroradiologist	0.29 ± 0.22	Precision: 0.26 ± 0.23 Recall: 0.61 ± 0.28
Winzeck et al. (28)	Multiscale U-net architecture trained with negative Dice score	MR-ADC, rCBF, rCBV, MTT, TMAX, TTP, Raw PWI and clinical information time-since-stroke, time-to-treatment, TICI and mRS scores	Final infarct lesions manually segmented on follow-up T2WI at 90 days by a neuroradiologist	0.31 ± 0.23	Sensitivity: 0.45 ± 0.31 Precision: 0.36 ± 0.27
Clèrigues et al. (29)	2D asymmetrical residual encoder–decoder CNN by using a more regularized network training procedure, symmetric modality augmentation and uncertainty filtering	CT-raw CTP series and CBF, CBV, MTT, Tmax	Infarct core manually segmented by a single investigator and then subjected to group review until acceptance	0.547 ± 0.242	Sensitivity: 0.609 ± 0.250
Ho et al. (30)	Unit CNN-contralateral model including modified input patches (patches of interest paired with contralateral patches), convolutional layer architecture and unit temporal filter learning	MR-PWI source image	Infarct lesions semi-automatically segmented on follow-up FLAIR at 3–7 days by a radiologist	NR	AUC: 0.871 ± 0.024 Precision: 0.222 Recall: 0.799
**Kasasbeh et al**. **(****31****)**	Feed-forward ANN	CT-rCBF, CBV, MTT, and Tmax	acute infarct lesions segmented on follow-up DWI at median time delay of 40.5 min	0.48 (IQR 0.23–0.70)	AUC: 0.85 Mean volume error: 13.8 ± 13.6 ml
Pérez Malla et al. (32)	DeepMedic model with PReLU activation using transfer learning, data augmentation and binary morphological post-processing operations	MR-ADC, MTT, and rCBF	Final infarct lesions manually segmented on follow-up T2WI at 90 days by a neuroradiologist	0.34	-
Robben et al. (33)	Fully convolutional network with PReLU activation	CT-native CTP, downsampled CTP, arterial input function and clinical data-time between stroke onset and imaging, time between imaging and the end of the mechanical thrombectomy, mTICI score and persistence of occlusion at 24 h	Infarct lesions semi-automatically segmented on follow-up NCCT at 1–5 days by an experienced reader	0.48	Mean absolute volume error: 36.7 ml
Winder et al. (34)	Random forest classifier	MR-ADC, distance to ischemic core, tissue type, anatomical location, CBV, MTT, Tmax, CBF and clinical data-NIHSS, age, sex, and time from symptom onset	Final infarct lesion manually segmented on FLAIR or DWI or NCCT at 5–7 days by an experienced medical expert	0.447 ± 0.247	-
Grosser et al. (35)	Random forest classifier trained by local and global approaches	MR-ADC, CBF, CBV, MTT, Tmax	Infarct lesions manually segmented on follow-up FLAIR at 1–7 days by 2 neurologists in consensus	0.353 ± 0.220	AUC: 0.859 ± 0.089 Sensitivity: 0.415 ± 0.231 Specificity: 0.964 ± 0.034
Grosser et al. (36)	XGBoost	MR-ADC, CBF, CBV, MTT, Tmax and voxel-wise lesion probabilities	Infarct lesions manually segmented on follow-up FLAIR within 7 days by 2 neuroradiologists in consensus	0.395 ± 0.229	AUC: 0.888 ± 0.101
Hu et al. (37)	Brain SegNet: a 3D dense segmentation network based on ResNet and trained with data augmentation and Focal loss	MR-TTP, Tmax, rCBV, rCBF, MTT, ADC	Final infarct lesions manually segmented on follow-up T2WI at 90 days by a neuroradiologist	0.30 ± 0.22	Precision: 0.35 ± 0.27 Recall: 0.43 ± 0.27
**Kim et al**. **(****38****)**	Random forest classifier	Features derived from MR-ADC and rTTP: range, mean, median, min, max, standard deviation, skew, kurtosis, 10 th percentile, 25 th percentile, 75 th percentile, and 90 th percentile	Infarct lesions manually segmented on follow-up DWI at 7 days	0.49 (IQR 0.37–0.59)	Unsuccessful recanalization: AUC: 0.746 ± 0.048 Mean volume error: −32.5 ml Successful recanalization: AUC: 0.764 ± 0.127 Mean volume error: 3.5 ml
Kumar et al. (39)	Classifier-Segmenter network, using a hybrid training strategy with a self-similar (fractal) U-Net model	MR-DWI, ADC, CBV, CBF, MTT, TTP, Tmax	Final infarct lesions manually segmented on follow-up T2WI at 90 days by a neuroradiologist	0.28 ± 0.22	Precision: 0.37 ± 0.29 Recall: 0.45 ± 0.34
**Pinto et al**. **(****40****)**	Two-branch Restricted Boltzmann Machine provides lesion and hemodynamics features from parametric MRI maps, then combined with parametric MRI maps and fed to a U-net using NReLU activation	MR-ADC, MTT, TTP, rCBF and rCBV	Final infarct lesions manually segmented on follow-up T2WI at 90 days by a neuroradiologist	0.38 ± 0.22	Precision: 0.41 ± 0.26 Recall: 0.53 ± 0.29
Qiu et al. (41)	Random forest classifier	Features derived from NCCT: Hounsfield units, bilateral density difference, hypoattenuation measurement, distance feature, atlas-encoded lesion location feature	Early infarct lesions manually segmented on follow-up DWI within 1 h	NR	Mean volume error: 11 ml
Wang et al. (42)	CNN model with a feature extractor, a pseudo-DWI generator and a final lesion segmenter using hybrid loss function	CT-CBF, CBV, MTT, Tmax and synthesized pseudo-DWI	Infarct core manually segmented by a single investigator and then subjected to group review until acceptance	0.54 ± 0.21	Precision: 51.20 ± 22.00 Recall: 64.20 ± 23.99
Yu et al. (17)	2.5D attention-gated U-Net using mixed loss functions	MR-DWI, ADC, Tmax, MTT, CBF, CBV	Final infarct lesions manually segmented on follow-up T2-FLAIR at 3–7 days by a neuroradiologist	0.53 (IQR 0.31–0.68)	AUC: 0.92 (IQR 0.87–0.96) Mean volume error: 9 ml (IQR −14ml−29ml)
**Benzakoun et al**. **(****11****)**	Gradient Boosting	MR-DWI, ADC, Tmax, MRR, CBF, CBV	Infarct lesions manually segmented on follow-up DWI around 24 h by a neuroradiologist	0.53 (IQR 0.29–0.68)	AUC: 0.98 (IQR 0.95–0.99) Mean volume error: 27.7 ± 40.3 ml
**Debs et al**. **(****43****)**	U-Net with multi-class Dice loss functions	MR-DWI, ADC, Tmax, CBF, CBV	Final infarct lesions semi-automatically segmented on follow-up T2-FLAIR at 6- or 30-day using intensity-based thresholding method	Reperfused: 0.44 ± 0.25 Non-reperfused: 0.47 ± 0.17	Reperfused: AUC: 0.87 ± 0.13 Precision:0.50 ± 0.27 Recall:0.50 ± 0.26 Non-reperfused: AUC: 0.81 ± 0.13 Precision: 0.49 ± 0.22 Recall: 0.52 ± 0.21
Hakim et al. (44)	3D multi-scale U-shape network with atrous convolution	CT-CTP source data, CBF, CBV, MTT, Tmax	Infarct core manually segmented by a single investigator and then subjected to group review until acceptance	0.51 ± 0.31	Mean absolute volume error: 10.24 ± 9.94 ml Precision: 0.55 ± 0.36 Recall: 0.55 ± 0.34
Hokkinen et al. (45)	3D CNN	CT-CTA source image	Infarct lesions manually segmented on follow-up CT with median time interval of 36 h	NR	Mean volume error: −16.3 ml
Hokkinen et al. (46)	3D CNN	CT-CTA source image	Infarct lesions manually segmented on follow-up CT or DWI within 5 days by a radiologist	NR	Mean volume error: 13.9 ± 12.5 ml
Klug et al. (47)	General linear regression model	CT-MTT, Tmax, CBF and CBV and multi-perfusion parameter analysis	Final infarct lesions segmented on T2-FLAIR within 10 days by 2 neuroradiologists	0.155	AUC: 0.89 Volume error: IVT: 4.6 ml (IQR 0.7–19.9), EVT: 32.8 ml (IQR 8.9–64.7)
**Kuang et al**. **(****13****)**	Random forest classifier	CT-average map, Tmax, CBF, CBV and clinical data-onset-to-imaging time, imaging-to-reperfusion time	PRoveIT study: infarct lesions manually segmented on follow-up DWI or NCCT by 2 experts in consensus; HERMES collaboration: infarct lesions automatically segmented followed by manual corrections	0.388 (IQR 0.192–0.541)	AUC: 0.81 ± 0.11 Volume error: −3.2 ml (IQR −16.7–6.1)
Modrau et al. (48)	Random forest classifier	MR-ADC, CBF, CBV, MTT, Tmax, tissue type probability, anatomical location, distance to the ischemic core and clinical data-age, sex, baseline NIHSS, time of stroke onset to medical application	Infarct lesions manually segmented on follow-up T2-FLAIR at 24 h	Theophylline subgroup: 0.40 ± 0.249 Placebo subgroup: 0.35 ± 0.243	
Pinto et al. (49)	2D U-Net with a data-driven branch computing spatio-temporal features from DSC-MRI	MR-DSC-MRI spatio-temporal information, Tmax, TTP, MTT, rCBV, rCBF, ADC	Final infarct lesions manually segmented on follow-up T2WI at 90 days by a neuroradiologist	0.31 ± 0.21	Precision: 0.29 ± 0.23 Recall: 0.63 ± 0.30
Qiu et al. (15)	Random forest classifier	Features derived multi-phase CTA: average and standard deviation of HUs across 3-phase CTA images, coefficient of variance of HUs in 3-phase CTA images, changing slopes of HUs between any two phases, peak of HUs in 3-phase CTA images, time of peak HU	Infarct lesions manually segmented on follow-up DWI/NCCT at 24/36h by 2 radiologists	0.247 (IQR 0.138–0.304)	Mean volume error: 21.7 ml
**Soltanpour et al**. **(****50****)**	MultiRes U-Net	CT-CBF, CBV, MTT, Tmax, contrast map, Tmax heatmap	Infarct core manually segmented by a single investigator and then subjected to group review until acceptance	0.68 ± 0.26	Sensitivity: 0.68 ± 0.15 Mean absolute volume error: 22.62 ± 7.3 ml
Vupputuri et al. (51)	MCN-DN: Multi-path convolution leveraged attention deep network with LReLU	MR-ADC, CBF, CBV, MTT, TTP	Final infarct lesions manually segmented on follow-up T2WI at 90 days by a neuroradiologist	0.47	Sensitivity:0.867 Specificity:0.972
**Yu et al**. **(****16****)**	Attention-gated U-Net with mixed loss functions	MR-DWI, ADC, Tmax, MTT, CBV, CBF and masks of Tmax (>6s) and ADC (620 × 10-6 mm2/s )	iCAS and DEFUSE-2 studies: final infarct lesions segmented on T2-FLAIR at 3–7 days; DEFUSE study: final infarct lesions segmented on T2-FLAIR at 30 days	0.57 (IQR 0.30–0.69)	AUC: 0.94 (IQR 0.89–0.97) Volume error: 0 ml (IQR -44–81)
He et al. (12)	2D U-Net with binary focal loss and Jaccard loss combined functions	CT-CBF, CBV, MTT, Tmax	Infarct lesions manually segmented on follow-up DWI/SWI or NCCT	0.61	AUC: 0.92 Sensitivity: 0.63 Specificity: 0.98 Absolute volume error: 26.5 ml (IQR 9.9–31.7)
**Lin et al**. **(****52****)**	R2U-RNet with residual refinement unit (RRU) activation and multiscale focal loss functions	CT-NCCT with intensity normalization and histogram equalization	Infarct lesion manually segmented on follow-up DWI within 7 days by a radiologist	0.54 ± 0.29	-
Shi et al. (53)	C^2^MA-Net: a cross-modal cross-attention network	CT-CBF, CBV, MTT, Tmax	Infarct core manually segmented by a single investigator and then subjected to group review until acceptance	0.48	Precision: 0.48 Recall: 0.59
**Zhu et al**. **(****54****)**	ISP-Net: a multi-scale atrous convolution with weighted cross entropy loss functions	CT-CTP source data, CBF, CBV, MTT, Tmax	Infarct lesions segmented on follow-up CT or DWI at 1-7 days	0.801 ± 0.078	AUC: 0.721 ± 0.108 Specificity:0.995 ± 0.002 Precision: 0.813 ± 0.066 Recall: 0.795 ± 0.115

*Y, Yes; N, No; NR, not reported; PReLU, parametric rectified linear unit; NReLU, noisy rectified linear unit; LReLU, leaky rectified linear unit; CTA, CT angiography; CTP, CT perfusion; DWI, diffusion-weighted imaging; ADC, apparent diffusion coefficient; CBF, cerebral blood flow; CBV, cerebral blood volume; MTT, mean transit time; TTP, time to peak; Tmax, time to maximum of the residue function; NIHSS, National Institute of Health stroke scale; mTICI, modified thrombolysis in cerebral infarction; mRS, modified Rankin scale; DSC, dice similarity coefficient; AUC, area under the receiving operator characteristic curve.*

*Studies included in the meta-analysis were presented in bold font*.

Thirteen studies adopted conventional ML algorithms including k-nearest neighbor classification (24), general linear regression (47), random forest (13, 15, 25, 34, 36, 38, 41, 48) and gradient boosting (11, 26, 36) classifiers. Twenty-five studies proposed DL-based approaches consisting of artificial neural network (ANN) (31) and various types of convolutional neural network (CNN) with some of the noteworthy popular architectures, including 2D and 3D U-Net (12, 16, 17, 27, 28, 39, 40, 43, 49, 50), residual network (ResNet) (12, 29, 37, 50), recurrent residual U-Net (R2U-Net) (52) and DeepMedic (32). Four studies applied modifications of the common rectified linear unit (ReLU) activation function for non-linear transformation after each convolution operation, including parametric ReLU, noisy ReLU, and leaky ReLU activation (32, 33, 40, 51). Given the class imbalance issue, 6 studies used hybrid loss function methods for target lesion segmentation (12, 16, 17, 29, 42, 52). Four studies introduced optimization strategies such as data augmentation for the training procedure (28, 29, 37, 39). The reference standard for model training was actual infarct lesion manually or semi-automatically segmented on follow-up CT or MR images with a wide-range time interval from 1 h to 90 days from baseline imaging.

Eleven studies used CT perfusion source data and parametric maps as model input for core infarct estimation (12, 13, 29, 31, 33, 42, 44, 47, 50, 53, 54), including one study generating a synthesized pseudo-DWI map based on CTP parametric maps (42). Five studies used source images and features derived from non-contrast CT (41, 52) and CT angiography (15, 45, 46). Twenty-two studies adopted different combinations of MRI sequences including T1WI, T2WI, diffusion and perfusion for infarct core prediction (11, 14, 16, 17, 24–28, 30, 32, 34–40, 43, 48, 49, 51). In addition to imaging data, 6 studies added clinical information into the model inputs, such as stroke severity quantified by the National Institutes of Health Stroke Scale [NIHSS] and modified Rankin Scale [mRS] scores, recanalization status assessed by modified Thrombolysis in Cerebral Infarction [mTICI] score and time variants of onset-to-imaging time and onset-to-treatment time (12, 23, 26, 41, 43, 45, 46).

Outcome measures were heterogeneous across studies. As infarct core estimation is a prediction segmentation task, model performance was commonly evaluated using the DSC score in all except 7 studies (14, 24, 26, 30, 41, 45, 46). Other metrics such as AUC, sensitivity, and specificity for classification results, accuracy, precision and recall for detection results, and a clinically intuitive metric of volume error were also employed and summarized in Table 1.

### Performance for Core Infarcted Tissue Prediction: A Meta-Analysis

Eleven studies were included in the meta-analysis (11, 13, 16, 25, 31, 38, 40, 43, 50, 52, 54). Methodological quality assessment of the included studies is shown in Table 2. Three studies clearly defined all three image sets of training, validation, and test (11, 16, 31). Only one study determined model performance using an external test set (13). Imaging data from one study were collected from four major manufacturers (44), five studies reported using single-vendor data (11, 25, 38, 40, 54), and others remained unknown (13, 16, 31, 43, 52). Although all the included studies clearly defined the validation methods, the relationship between the number of training images and model performance (i.e., sample size estimation) was not carefully evaluated. All studies described the data pre-processing procedure, trained their models using acceptable reference standards, and demonstrated the predictive performance assessed by multiple performance metrics. Algorithms from two studies were partially publicly available via the website of GitHub (11, 40).

**Table 2:** Methodological quality assessment of the included studies.

**Quality assessment items**	**McKinley (25)**	**Kasasbeh (31)**	**Kim (38)**	**Pinto** **(40)**	**Benzakoun (11)**	**Debs** **(43)**	**Kuang (13)**	**Soltannpour** **(50)**	**Yu (16)**	**Lin** **(52)**	**Zhu (54)**
Are all three image sets (training, validation, and test sets) defined?	N	Y	N	N	Y	N	N	N	Y	N	N
Is an external test set used for final statistical reporting?	N	N	N	N	N	N	Y	N	N	N	N
Have multivendor images been used to evaluate the AI algorithm?	N	U	N	N	N	U	U	Y	U	U	N
Are the sizes of the training, validation and test sets justified?	U	U	U	U	U	U	U	U	U	U	U
Was the AI algorithm trained using a standard of reference that is widely accepted in our field?	Y	Y	Y	Y	Y	Y	Y	Y	Y	Y	Y
Was preparation of images for the AI algorithm adequately described?	Y	Y	Y	Y	Y	Y	Y	Y	Y	Y	Y
Were the results of the AI algorithm compared with radiology experts and/or pathology?	Y	Y	Y	Y	Y	Y	Y	Y	Y	Y	Y
Was the manner in which the AI algorithms makes decisions demonstrated?	Y	Y	Y	Y	Y	Y	Y	Y	Y	Y	Y
Is the AI algorithm publicly available?	N	N	N	Y	Y	N	N	N	N	N	N

*Y, yes; N, no; U, unknown*.

**Figure 2:**
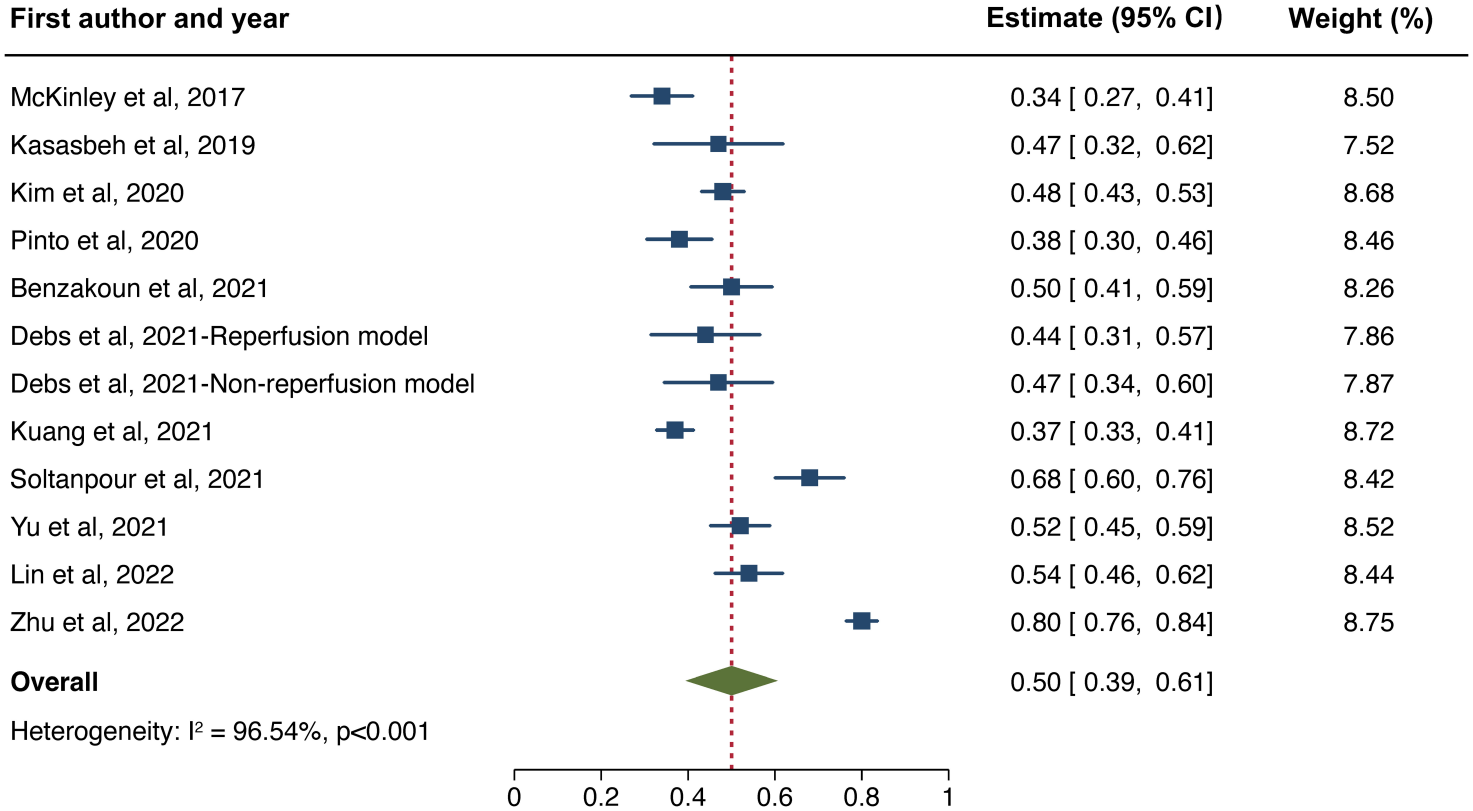
Forest plot of the included studies that assessed the performance of infarct tissue outcome prediction. Forest plot shows that the dice similarity coefficient (DSC) representing the performance of the machine learning-based approaches for final infarct prediction centers around 0.50 with a 95% confidence interval (CI) ranging from 0.39 to 0.61.

**Figure 3:**
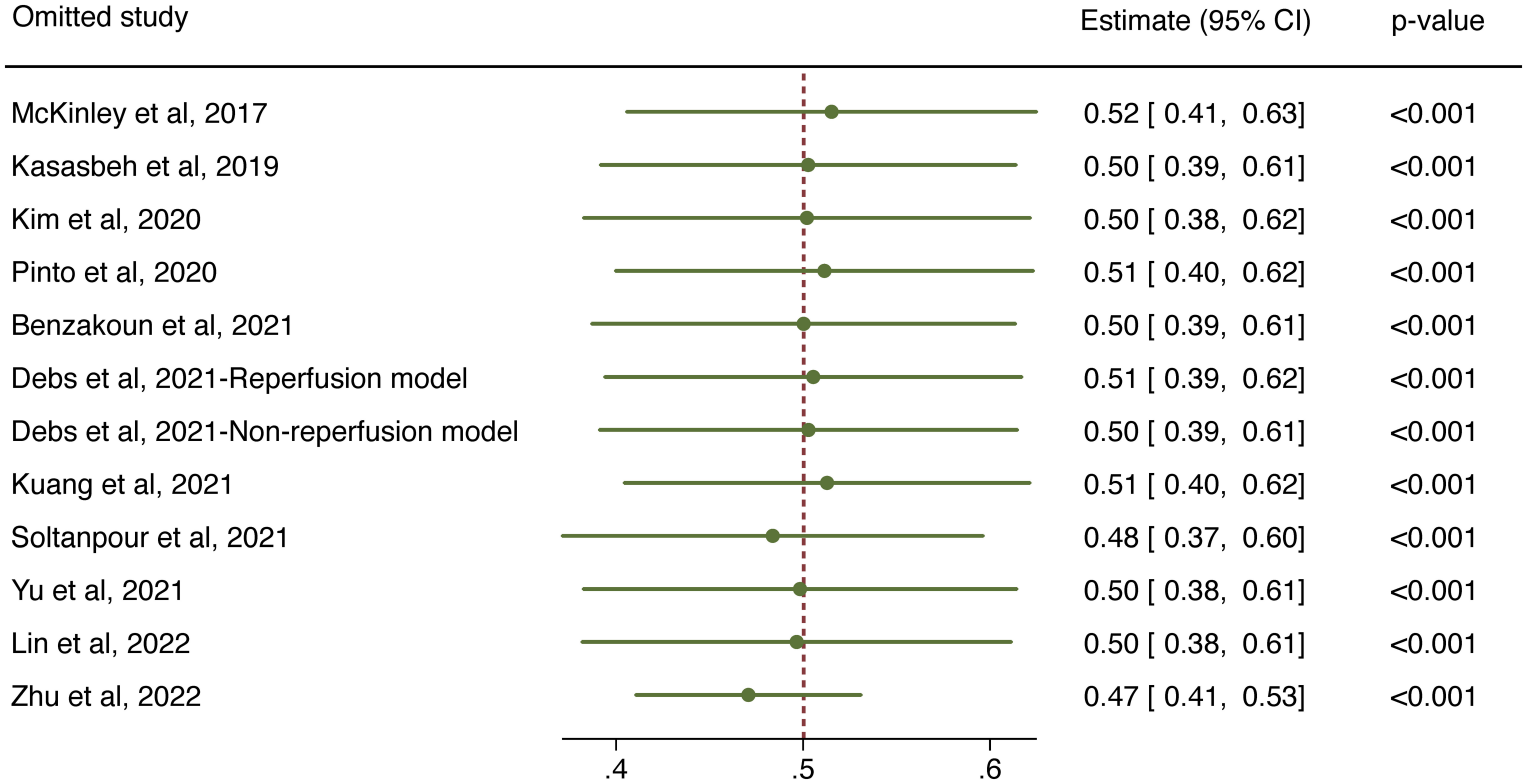
Sensitivity analysis for the overall predictive performance using one-study-removed method.

**Figure 4:**
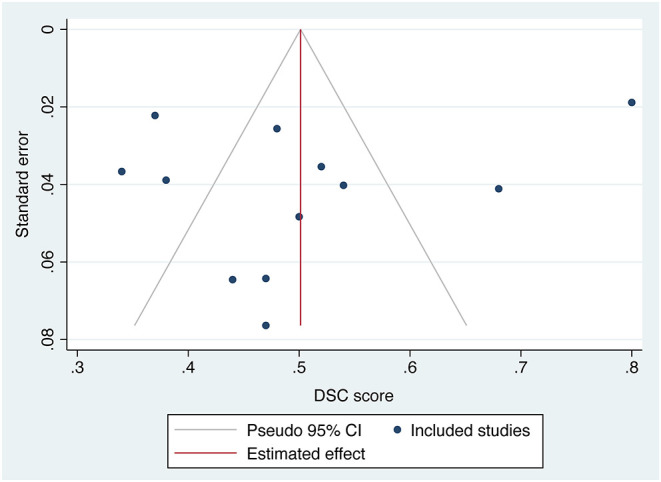
Funnel plot of the included studies. The effect size of mean dice similarity coefficient (DSC) score was displayed on the horizontal axis. Standard error was plotted on the vertical axis.

The overall performance of 11 predictive models is presented in Figure 2. The pooled DSC score was 0.50 (95% CI 0.39–0.61). The value of Cochrane *Q* test *p* < 0.001 and Higgins *I*^2^ of 96.5%, indicating high heterogeneity across the included studies. We conducted a sensitivity analysis by removing one study at each step (Figure 3). The adjusted overall DSC score ranged from 0.47 (95% CI 0.41–0.53) after removing the study by Zhu et al. (54) to 0.52 (95% CI 0.41–0.63) after removing the study by McKinley et al. (25). Publication bias assessed by graphic funnel plot showed an asymmetrical shape, and not all studies were plotted within the area under the curve of the pseudo-95% CI, indicating the potential publication bias among included studies (Figure 4). Egger's test showed no statistically significant publication bias (*p* = 0.565).

**Figure 5:**
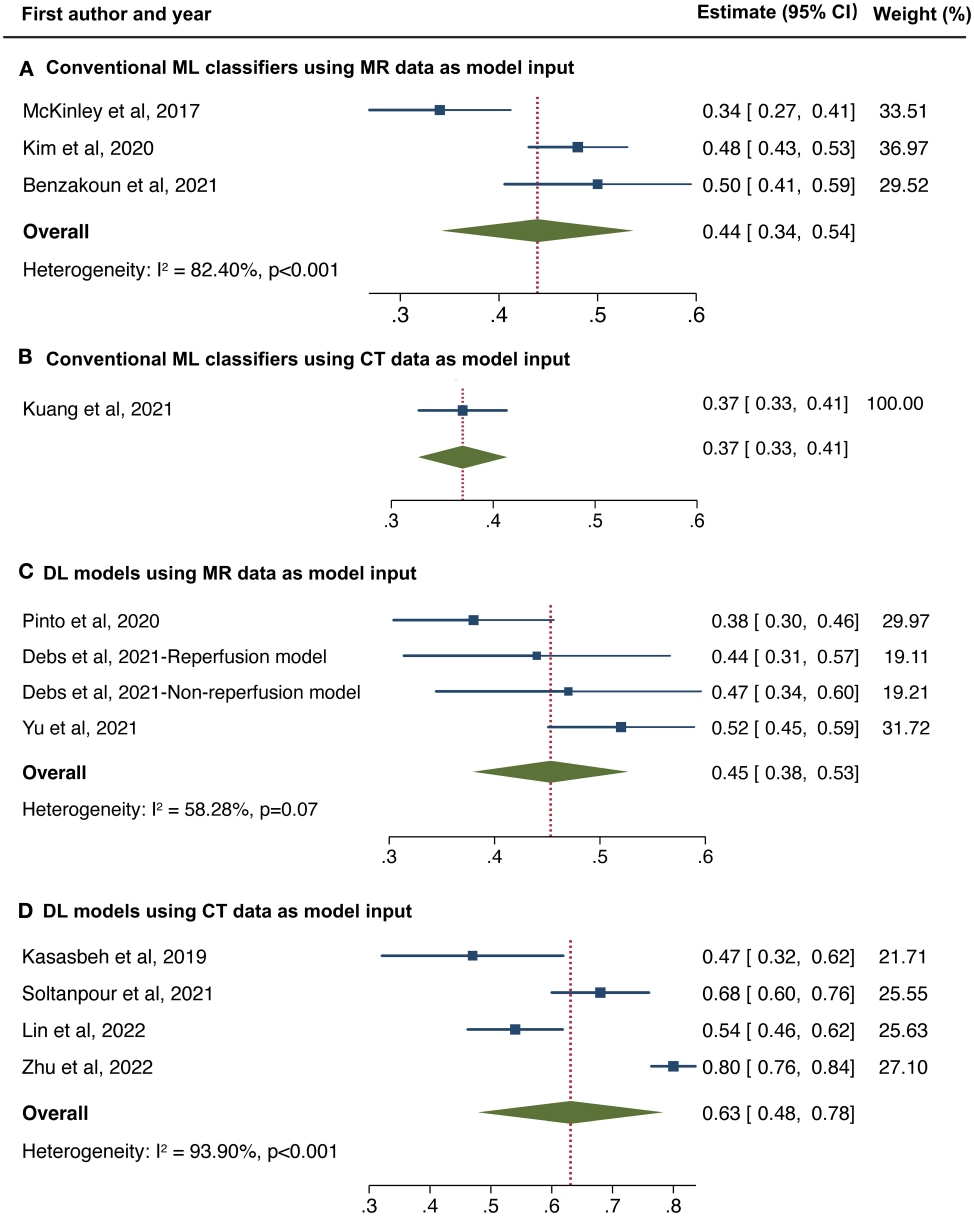
Forest plot of subgroup analyses in conventional machine learning (ML) classifiers using MR data **(A)** and CT data **(B)** as model input, and deep learning models using MR data **(C)** and CT data **(D)** as model input, respectively.

We made further subgroup analyses by algorithm types and imaging modality. Forest plot for each subgroup is depicted in Figure 5. For studies applying conventional ML classifiers, the pooled DSC score was 0.44 (95%CI 0.34–0.54) for models inputting MR data (11, 25, 38) and 0.37 (95%CI 0.33–0.41) for a single model using CT data as a reference (13). For studies developing DL-based approaches, the pooled DSC was 0.45 (95%CI 0.38–0.53) for models inputting MR data (16, 40, 43) and 0.63 (95%CI 0.48–0.78) when using CT data (31, 50, 52, 54). Sustained high heterogeneity was observed in the subgroup of DL models combined with CT data (Higgins *I*^2^ 93.9%, *p* < 0.001). Sensitivity analysis revealed that after removing one study (54), the adjusted pooled DSC score was 0.59 (95%CI 0.54–0.64), with a downward trend of heterogeneity (Higgins *I*^2^ of 77.3%, *p* = 0.012).

## Discussion

In this study, we reviewed the performance of ML-based approaches for final infarct lesion prediction from acute stroke imaging. The overall predictive performance of ML algorithms was moderate with a pooled DSC score of 0.50 (95% CI 0.39–0.61, Higgins *I*^2^ = 96.5%, *p* < 0.001). Subgroup analyses indicated that the DL-based models outperformed the conventional ML classifiers with the best performance observed in DL algorithms combined with pre-processing CT data. Although high heterogeneity was present across studies, current ML algorithms still showed promising performance for ischemic tissue outcome prediction from baseline imaging.

Estimating the final infarct lesion from baseline imaging is complex due to the heterogeneity of lesion shape, location, and progression. The aim of ML applications is to exact the maximum amount of predictive power from the available multi-modality imaging information, where conventional thresholding methods seem inadequate (10). A few studies validated their proposed ML-based approaches compared to conventional thresholds for core infarcted tissue delineation and showed significant improvement in measurement results using ML-based methods (11, 13–15, 17). For instance, when training an attention-gated U-Net with baseline MR diffusion and perfusion parameters, the prediction model outperformed the ADC <620 × 10^−6^ mm^2^/s threshold, with more precise segmentation (DSC score 0.53 vs. 0.45), higher discriminating power (AUC 0.92 vs. 0.71) and smaller volume error (median 9 vs. 12 ml) (14). Such strategies take advantage of the data-processing ability of ML algorithms to provide rapid and reliable assessment, which is promising to support clinical management.

Conventional ML classifiers included linear regression and decision trees. Grosser et al. compared the performance of 3 classical ML algorithms for infarct core estimate and revealed that decision trees (random forest and gradient boosting) performed better than linear regression model (36), indicating the necessity of using non-linear algorithms for stroke prediction segmentation. In the 2017 ISLES challenge, where uniform pre-processing data for model training and test were provided, almost all top-ranking teams employed DL algorithms instead of ML classifiers (28). Our finding was consistent with the results of ISLES challenge 2017, which indicated the advantages of using DL algorithms for final infarct prediction. However, a recent study held contradictory results that a U-net model performed less well than two decision tree classifiers (DSC score 0.48 vs. 0.53 and 0.51) (11). A possible reason was the relatively small sample size for fully training a DL algorithm. The inherent data-dependent characteristic of DL algorithms meant that once trained on sufficient data, the model performance would continue to improve, while classical ML approaches tend toward stability. Moreover, most well-performed DL models were customized on the baseline architectures. Innovative modifications of algorithm architectures and training strategies would further improve the predictive performance of DL models.

Most of the previous works have chosen MR images for model training, given the high tissue contrast and the sensitivity of MR diffusion for infarct core detection. Our study revealed that the DSC score of MR-input models stabilized at around 0.45, either using ML classifiers or DL models. In clinical practice, CT is more widely available for acute stroke triage, detecting large vessel occlusion, and selecting candidates for revascularization (8, 55). Studies developing models training on CT perfusion data appeared late yet achieved comparable or better performance than MR-input models (12, 29, 31, 33, 42, 44, 47, 50, 52, 53). One study employing a random forest classifier using features extracted from multi-phase CT angiography presented less satisfactory performance with a DSC score of 0.22 (15). However, a more recent study based on the R2U-RNet algorithm using non-contrast CT data with intensity normalization and histogram equalization showed promising performance with a DSC score of 0.54 (52). Theoretically, different imaging modalities and parameters provide complementary information, and thus the combination of multimodal imaging data with reasonable pre-processing would enhance the overall predictive performance. In addition, several approaches incorporated clinical data such as stroke severity, reperfusion status, and time variants (13, 28, 33, 34, 48). Multi-dimensional input information consisting of imaging and non-imaging data is expected to establish better prediction models, which is a direction of future research.

In our study, we have chosen the DSC score as the primary performance metric, a commonly used volume-based metric containing lesion size and location information for target segmentation. Other segmentation metrics, such as Jaccard index was less reported in this research field, and Hausdorff distance and surface distance were distance-based metrics that were less optimal for final infarct lesion prediction (56). Although ROC is more familiar in diagnostic accuracy studies, its efficacy has been challenged for class imbalance tissue, such as infarct core prediction. Large numbers of “healthy” voxels would lead the AUC values to a high level and reduce its discriminating power. From a clinical standpoint, we included the volume error as a secondary performance metric, which enabled intuitive assessment of the size differences between prediction results and reference standards, as the estimate of core infarct volume was critical to identify eligible patients who would benefit from treatment in the late time window.

Although ML-based approaches provided promising results for final infarct lesion prediction, there is still no wide acceptance and implementation in clinical practice. Most of the proposed models were trained on datasets with small sample size, which was deemed insufficient to train an ML algorithm (especially a DL algorithm), leading to an overall moderate predictive performance. Many studies validated using the k-fold cross-validation method to provide an unbiased evaluation with small sample size. However, the real predictive performance would be overestimated without an independent external validation (57). Another limitation was data heterogeneity, as models trained on single-center cohort using single-vendor data would reduce the model generalizability. One study validating their approach on an external cohort indicated less satisfactory performance with a median DSC score of 0.39 (13). There is an emerging trend to build up large multi-vendor, multi-institution diagnostic datasets with initial implementation on chest X-ray data (56). A similar dataset for stroke lesion segmentation would be helpful. In addition, a standardized methodologic procedure is also warranted including the definition of the clinical cohort, imaging protocols, reference standard, model training and validation process, and clinical evaluation of model performance.

Our study has several limitations. First, the heterogeneity was high across studies due to the differences in the study cohorts, algorithm types, and input parameters. We made sensitivity analyses and found no obvious deviation of the adjusted effect size from the main effect size. We also conducted subgroup analyses to explain the heterogeneity and found a downward trend of heterogeneity in the subgroup analyses. However, re-evaluation of the overall model performance is needed as more relevant, intensive studies accumulate. Second, as an emerging field of artificial intelligence in imaging, there was no consensus on the reporting standards. Therefore, 13 studies were excluded before the meta-analysis because of lacking definition of image sets or results of DSC scores, which might result in an incomplete assessment of available studies. Third, although the publication bias examined by Egger's test was not significant, the funnel plot showed an asymmetrical shape. We excluded 14 studies due to dataset duplicates or overlapping to avoid affecting the overall pooled effect size. It might contribute to the risk of publication bias.

## Conclusion

In this study, we conducted a systematic review and meta-analysis of current studies using ML algorithms for infarct core prediction. Despite the heterogeneity across studies, the overall performance of ML-based predictive methods is moderate but promising, with better predictive performance presented in the DL-based approaches. However, before well integrated into clinical stroke workflow, future studies are suggested to train ML-based approaches on large-scale, multi-vendor data, validate on external cohorts and adopt formalized reporting standards for improving model accuracy and robustness.

## Data Availability Statement

The original contributions presented in the study are included in the article/supplementary material, further inquiries can be directed to the corresponding author/s.

## Author Contributions

XW conducted the literature search. XW, YF, and NZ participated in study selection and data collection. XW and YF analyzed the data and drafted the manuscript. NZ, JL, YD, and BY revised the manuscript for important intellectual content. BY designed the review protocol and he was the study guarantor. All authors approved the final version of the manuscript.

## Funding

This study was supported by grant 2019-BS-267 from the Project of Scientific Research Foundation for the Ph.D. of Liaoning Province, and by grant 20-205-4-086 from the Project of Natural Science Foundation of Shenyang.

## Conflict of Interest

The authors declare that the research was conducted in the absence of any commercial or financial relationships that could be construed as a potential conflict of interest.

## Publisher's Note

All claims expressed in this article are solely those of the authors and do not necessarily represent those of their affiliated organizations, or those of the publisher, the editors and the reviewers. Any product that may be evaluated in this article, or claim that may be made by its manufacturer, is not guaranteed or endorsed by the publisher.
